# Spectral CT Imaging of Prosthetic Valve Embolization after Transcatheter Aortic Valve Implantation

**DOI:** 10.3390/diagnostics13040678

**Published:** 2023-02-11

**Authors:** Tommaso D’Angelo, Giampiero Vizzari, Ludovica R. M. Lanzafame, Federica Pergolizzi, Silvio Mazziotti, Michele Gaeta, Francesco Costa, Gianluca Di Bella, Thomas J. Vogl, Christian Booz, Antonio Micari, Alfredo Blandino

**Affiliations:** 1 Diagnostic and Interventional Radiology Unit, BIOMORF Department, University Hospital Messina, 98124 Messina, Italy; 2 Department of Radiology and Nuclear Medicine, Erasmus MC, 3015 GD Rotterdam, The Netherlands; 3 Cardiology Unit, BIOMORF Department, University Hospital Messina, 98124 Messina, Italy; 4 Division of Experimental Imaging, Department of Diagnostic and Interventional Radiology, University Hospital Frankfurt, 60590 Frankfurt am Main, Germany

**Keywords:** aortic valve stenosis, heart valve prosthesis implantation, transcatheter aortic valve implantation, embolization, computed tomography angiography

## Abstract

Transcatheter heart valve (THV) embolization is a rare complication of transcatheter aortic valve implantation (TAVI) generally caused by malpositioning, sizing inaccuracies and pacing failures. The consequences are related to the site of embolization, ranging from a silent clinical picture when the device is stably anchored in the descending aorta to potentially fatal outcomes (e.g., obstruction of flow to vital organs, aortic dissection, thrombosis, etc.). Here, we present the case of a 65-year-old severely obese woman affected by severe aortic valve stenosis who underwent TAVI complicated by embolization of the device. The patient underwent spectral CT angiography that allowed for improved image quality by means of virtual monoenergetic reconstructions, permitting optimal pre-procedural planning. She was successfully re-treated with implantation of a second prosthetic valve a few weeks later.

**Figure 1:**
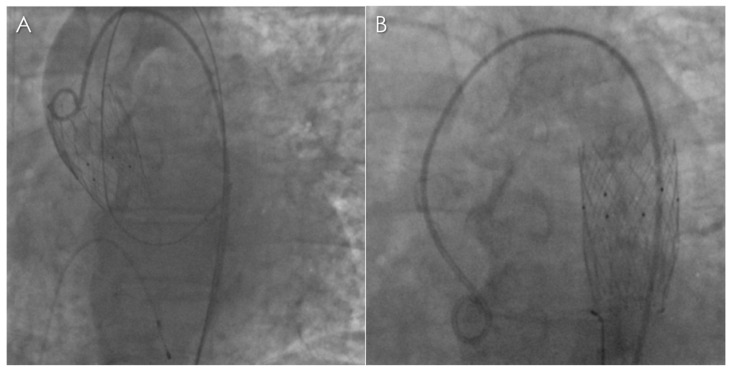
A 65-year-old female patient with medical history of diabetes, dyslipidemia, arterial hypertension, chronic obstructive pulmonary disease and class III obesity (BMI > 40), symptomatic for angina and dyspnea on mild effort underwent echocardiography and was diagnosed with severe aortic stenosis (mean gradient: 44 mmHg; maximum gradient: 75 mmHg; aortic jet velocity: 4.6 m/s; aortic valve area: 0.9 cm²). The therapeutic options for stenosis management included surgical aortic valve replacement and transcatheter aortic valve implantation (TAVI). Evaluation of the risk profile by The European System for Cardiac Operative Risk Evaluation (EuroSCORE II) [1] revealed an intermediate risk of in-hospital mortality. However, the decision to opt for the endovascular approach was taken due to patient’s severe obesity [2]. Prior to prosthetic valve implantation, the patient underwent pre-procedural planning computed tomography angiography (CTA) in another hospital on a conventional CT scanner. TAVI was performed with left transfemoral access. A 27 mm NVT Allegra aortic self-expanding transcatheter heart valve (THV) with bovine pericardial tissue was deployed. Unfortunately, the procedure was complicated by valve embolization, likely due to it being oversize (**A**). The embolized valve was therefore snared and stably anchored into the proximal descending thoracic aorta (**B**).

**Figure 2:**
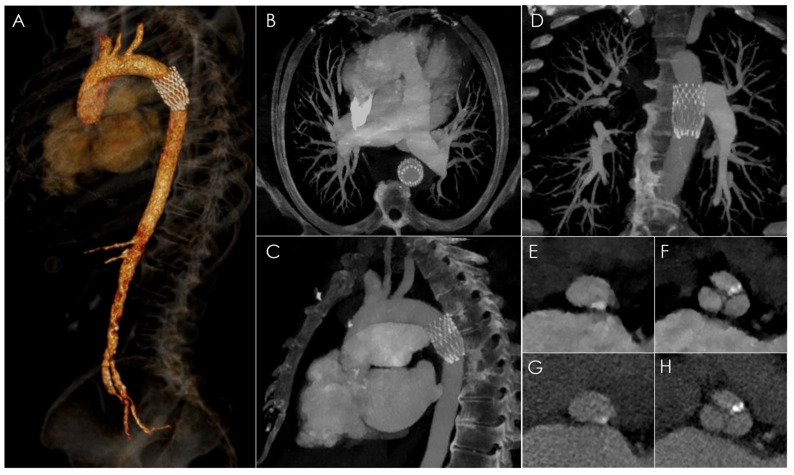
The patient was referred to our radiological department and underwent CTA on a dual-layer spectral CT scanner (IQon Spectral CT, Philips Healthcare, Best, The Netherlands) to rule out potential aortic complications, and to reassess the aortic valve annular size prior to a second TAVI procedure. A CT scan was performed in supine position, with acquisition of an ECG-synchronized dataset of the aortic root and heart followed by a CT Aortogram. The cumulative effective dose was 29 mSv. Fifty milliliters of iodinated contrast agent (Iomeron 400 mgI/mL, Bracco, Milan, Italy) was intravenously administered at a flow rate of 5 mL/s by antecubital access. Volumetric (**A**) and MIP (**B**–**D**) images based on 40 keV monoenergetic reconstructions (MonoE) allowed an increase in image quality by improving the contrast-to-noise ratio [3,4,5,6,7,8,9]. The images confirmed the dislocated prosthesis in the proximal descending thoracic aorta, but no aortic dissection, thrombosis or hematoma was present. MPR images along the aortic annulus and the sinus of Valsalva (**E**,**F**) allowed for a superior definition of anatomical sizing compared to the respective conventional images (**G**,**H**). The patient was indicated for a smaller valve prosthesis than the previous one.

**Figure 3:**
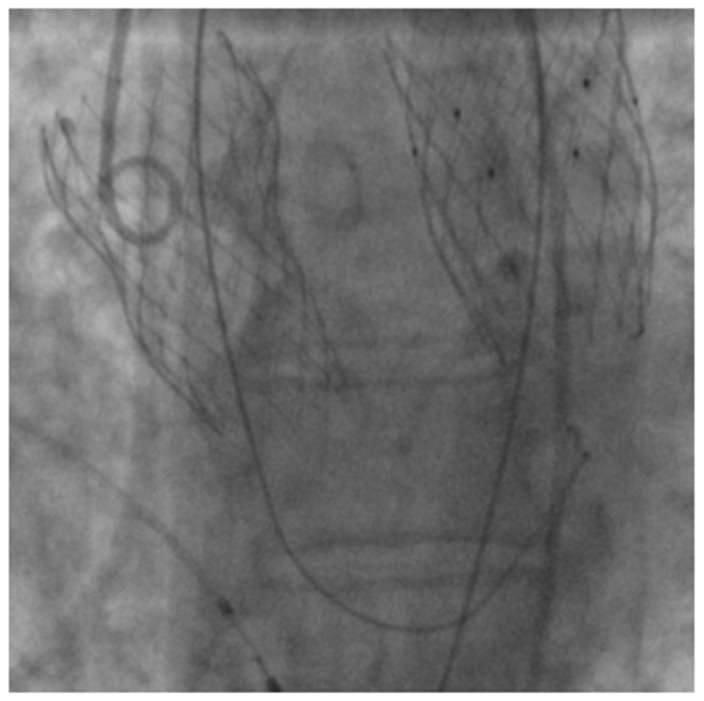
The second attempt of TAVI was performed using a 26 mm Medtronic Evolut R self-expanding transcatheter aortic valve with nitinol frame and a porcine pericardial tissue. The procedure was successful and the patient had no complications.

## Data Availability

No new data were created or analyzed in this study. Data sharing is not applicable to this article.
